# Is Searching for Meaning in Life Related to Civic Engagement?: Individual- and Society-Level Moderators

**DOI:** 10.3389/fpsyg.2019.01334

**Published:** 2019-06-26

**Authors:** Li Lin

**Affiliations:** Department of Applied Social Sciences, The Hong Kong Polytechnic University, Kowloon, Hong Kong

**Keywords:** search for meaning in life, civic engagement, values, power distance, cross-national study

## Abstract

Going beyond previous research suggesting that the search for meaning in life (hereafter “search for meaning”) is associated with civic engagement, this study investigated the moderating effects of personal and cultural values on the associations between the search for meaning and two kinds of civic engagement (i.e., pro-environmental engagement and political engagement). Based on the dataset of the sixth wave of the World Values Survey (WVS), multi-level analyses showed that the association between the search for meaning (in terms of thinking about meaning and purpose of life) and pro-environmental engagement was stronger when people held stronger values of openness to change (vs. conservation) and prioritized environmental wellness. The association between the search for meaning and political engagement was stronger when people endorsed stronger values of openness to change, showed a greater interest in politics and attributed greater importance to politics. At the society level, the association between the search for meaning and civic engagement was stronger in societies with a lower power distance. Implications for individual differences of the meaning search are discussed.

## Introduction

The search for meaning in life (hereafter “search for meaning”) is conceptualized as the degree to which people desire and endeavor to construct or enhance their comprehension of life meaning, the significance of their lives, and purpose in their lives (Steger et al., 2006). It is not equivalent to having a meaningful life, which denotes a subjective experience that life is meaningful. Instead, it pertains to a process in which people cognitively or behaviorally explore how they can make their lives more meaningful, significant and purposeful (Steger et al., 2008a). Most people cognitively and behaviorally engage in the quest for meaning in life (Frankl, 1959; Heine et al., 2006; Benson et al., 2012), but what will they do when they are searching for meaning? Previous studies (Scales et al., 2014; van Tilburg and Igou, 2017) suggest that people who have greater engagement in the meaning search are more likely to take up civic actions such as joining pro-environment activities or donating to charity. However, civic engagement, broadly defined as participation in activities or events aimed at addressing issues of public concern to promote the quality of life in a given community or society (Brooks, 2017), is apparently not the only action that people will undertake to achieve a greater sense of meaning. Different individuals may prefer different means to enhance their meaningfulness. Therefore, identifying the individuals who would be more likely to use civic engagement to fulfill their life meaning becomes an important yet unresolved issue.

This study attempted to address this issue with reference to meaning regulation theories (Heine et al., 2006; van Tilburg and Igou, 2011). I contend that the likelihood that meaning searchers take civic actions depends on their personal values and the cultural values upheld by their society. I tested this proposition using the database of the sixth wave of the World Values Survey (WVS) (Inglehart et al., 2014). The findings of this study shed light on individual differences in behavioral orientation during human’s search for meaning.

### The Search for Meaning and Civic Engagement

Pragmatic meaning regulation theory (van Tilburg and Igou, 2011) suggests that when people are more eager to regain or enhance their sense of meaning, they are more attuned to potential behavioral strategies that can regulate their meaning, and they subsequently engage in specific behavior that allows them to maintain or increase meaningfulness. Such behavior is called “meaningful behavior.”

Previous research suggests that civic engagement probably represents a meaningful behavior. When young people (aged 12–24) showed stronger attempts to discover meaning, such as thinking about “big questions” (e.g., “Why we are here?”), they were more likely to conduct voluntary service and pro-environmental behavior (Scales et al., 2014). van Tilburg and Igou (2017) found that participants with stronger motivation to boost their sense of meaning expressed stronger intention of civic engagement. van Tongeren et al. (2016) also found higher levels of prosociality (i.e., disposition to help others) among people who encountered (experimentally induced) threats to meaning relative to those who received neutral stimulation, indicating that people’s needs to restore meaning enhance their willingness to contribute to others’ welfare.

Civic engagement presumably enhances people’s sense of belonging, self-esteem, sense of control, and symbolic immortality, four key sources of meaning in life (Heine et al., 2006). It is a process that connects individuals with common interests and values to strive for a common good. This process presumably strengthens people’s social connections with people they are working with and the communities they are working for Albanesi et al. (2007). The connection between people and their external world informs them about their positions in the world, thus providing them with a sense of meaning. Furthermore, social connection and identification with a social group can augment self-esteem, sense of control and even symbolic immortality (van Tilburg and Igou, 2011; Stavrova and Luhmann, 2016). Being engaged in and accepted by a larger social group likely boosts people’s sense of self-worth, making them feel that their existence is significant and that life is worth living (Stavrova and Luhmann, 2016). Additionally, group identification strengthens individuals’ sense of control because it makes them feel that they are capable of achieving desired outcomes (Greenaway et al., 2015). When people have personal control over their lives and can predict what will happen, they likely perceive more meaningfulness. Finally, as suggested by the terror management theory (Greenberg and Kosloff, 2008), fighting for one’s values and worldviews is a defense mechanism that helps people overcome the anxiety caused by the meaninglessness of their existence. People who are involved in civic actions usually adhere to and fight for a common value, and thus, they likely gain a sense of symbolic immortality.

Supporting evidence can be found from quantitative and qualitative studies. For example, participation in civic activities has been found to be associated with a stronger sense of belonging toward community (e.g., Ohmer, 2007; Talò et al., 2014). Besides, considerable studies have revealed that active civic participants tend to report greater self-esteem, self-efficacy, and empowerment than those non-engaged ones, feeling that they are more competent and capable to control their lives (e.g., Zimmerman and Rappaport, 1988; Pancer et al., 2007; Brown et al., 2012). Additionally, studies have suggested that social actions done for the sake of others enhance one’s meaningfulness through improving his/her relationship with others (van Tongeren et al., 2016) and fulfilling his/her needs for autonomy and competence (Martela and Ryan, 2016). Civic engagement, as one of social actions aimed at addressing welfare of others or groups, presumably enables people to reach their meaningfulness. Altogether, civic engagement is deemed a potentially meaningful behavior.

### Who Prefers Civic Engagement During the Meaning Search?

Civic engagement is not the only action that potentially contributes to meaningfulness. People are flexible and instrumental during meaning regulation (Heine et al., 2006). There are individual differences in how people restore and enhance their sense of meaning. For example, some prefer strengthening connection with others while some prefer striving for personal excellence. Identifying who is more likely to fulfill life meaning by civic engagement contributes to deepening our understanding of how people select their behavior when they are searching for meaning.

Before conducting a meaningful behavior, people need to judge the meaningfulness of this behavior (i.e., the extent to which it can enhance their meaningfulness), and they prefer to conduct behavior that has the greatest potential to enhance their meaningfulness. Values, as “a stable meaning-producing superordinate cognitive structure” (Rohan, 2000, p. 257), probably influence people’s judgment about the meaningfulness of a certain behavior (van Tilburg and Igou, 2013). Values determine what is desirable and important in one’s life, which provide people with a reference to select and justify their behaviors across circumstances (Schwartz, 2012). Accordingly, people often regard behavior that can fulfill their personal values to be more meaningful because value-expression and value-attainment enhance one’s sense of self-worth and personal growth (van Tilburg and Igou, 2013). These attainments are conducive to meaning in life (see Steger et al., 2006). Accordingly, people who value civic engagement are more likely to perceive it to be more meaningful and thus engage in civic actions to enhance their meaningfulness.

Furthermore, the meaningfulness of behavior is better understood as a function of both individual and contextual characteristics because this perception is not only subjective but also contextually sensitive (van Tilburg and Igou, 2013). A behavior may be considered meaningful in one context but not in others. People’s behavioral orientations probably depend on shared values, which vary across contexts. Thus, cultural values, which are “the implicitly or explicitly shared abstract ideas about what is good, right, and desirable in a society” (Schwartz, 1999, p. 25), warrant scrutiny. Dissimilar to personal values, cultural values are evolved in the process of confronting basic challenges in societies at large posed by social and ecological environments, and societies vary in the basic challenges faced as well as the ways to fight off them; thus rendering diversity of cultural orientations (Schwartz, 2006). These cultural orientations shape institutions and organizations of a society, which further affect individuals’ minds and actions. Briefly, cultural values provide a shared reference for judging whether a certain behavior is desirable and meaningful within a society, thereby guiding society members’ decisions and actions (Roccas and Sagiv, 2010). Accordingly, it is expected that people who live in a society that upholds civic engagement are more likely to perceive civic actions to be more meaningful and take such action for the sake of their meaningfulness.

To summarize, when people have stronger engagement in the search for meaning, they are more likely to engage in behavior that is personally valued or culturally valued, as they consider such behavior more meaningful. In the case of civic engagement, when people highly value the welfare of the community, society or earth and aim to improve this welfare through civic engagement, or when the culture encourages people to take actions to improve this welfare, their search for meaning are likelier to make them participate in civic activities.

### The Current Study

The purpose of the current study is to fill a major gap in the research regarding the individual differences in behavioral orientation during the search for meaning. As a conceptual replication of previous research (Scales et al., 2014; van Tilburg and Igou, 2017), this study examined whether individuals’ search for meaning increases their civic engagement in two domains, political engagement, and pro-environmental engagement. I expected that the search for meaning would be positively associated with both kinds of civic engagement (Hypothesis 1). Furthermore, extending previous research, I further tested a proposition contending that when people search for meaning, they are more likely to conduct meaningful behavior that is consistent with their personal values and cultural values. At the individual level, values can be categorized into two dimensions according to their motivation type (Schwartz, 2012). The first dimension contrasts “openness to change” with “conservation.” The second dimension contrasts “self-transcendence” with “self-enhancement.” Stronger endorsement of openness renders people perceive making changes to community and society and promoting public welfare (which are often novel and stimulating) to be more desirable than preserving existing or traditional ideas, thus strengthening the linkage between the search for meaning and civic engagement. Values of self-transcendence (vs. self-enhancement) may also strengthen the linkage because this dimension of values emphasizes the welfare and interests of others over personal interests such as power and achievement. In addition to general values, domain-specific values also guide people to partake in a particular civic activity. For example, if one values environmental protection or politics, he or she may be more likely to search for meaning by engaging in pro-environmental activities or political activities. Therefore, this study tested the moderating roles of both general values orientation and specific values in the associations between the search for meaning and civic engagement.

At the society level, this study tested a dimension of cultural values: power distance. Power distance refers to the degree to which members in a given society or institution expect or accept an unequal power distribution in their social units (Hofstede, 1980). It is a societal level construct that reflects the strength of social hierarchy in a given society. In a society with a higher power distance, people tend to regard the inequality of social power to be more legitimate and the social hierarchy to be more justifiable; in a society with a lower power distance, shared authority is more common, and people are less tolerant of unequal power distribution. Culture is a shared meaning system that shapes the values shared by the majority of the residents within a society. In a society with a higher power distance, people are more likely to believe that public issues (e.g., environmental protection and poverty) are handled by power holders such as the government rather than non-power-holding members of society and that people should act according to their defined social positions (Winterich and Zhang, 2014). Therefore, interfering in public issues is not desirable, and civic engagement may not be meaningful to most individuals. In contrast, in a society with a lower power distance, people are more likely to believe that they share the responsibility for public issues and to value individual endeavors to fight public problems. Studies have found that people report lower levels of political engagement (Cohen and Valencia, 2008) and environmentalism (Chan et al., 2019) in societies with a higher power distance, suggesting that power distance is a cultural factor relevant to civic engagement. Accordingly, it is expected that civic engagement is likely perceived as more meaningful in a society with a lower power distance, and people searching for meaning are more likely to demonstrate civic engagement in such a society.

**FIGURE 1:**
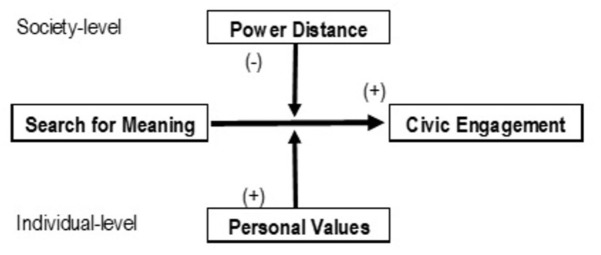
Illustration of hypothesized model.

Altogether, I expected to observe moderating effects of personal values and cultural values on the relationship between the search for meaning and civic engagement (see Figure 1). At the individual level, this relationship was expected to be stronger when people hold stronger values of openness to change or self-transcendence (Hypothesis 2a and 2b). In addition, the relationship between the search for meaning and political engagement (pro-environmental engagement) was expected to be stronger when people more strongly value politics (environmental protection) (Hypothesis 3). At the society level, this relationship was expected to be stronger when the power distance is lower (Hypothesis 4).

## Materials and Methods

### Participants

The analyses were primarily based on the cross-national representative samples examined in the sixth wave of the WVS (Inglehart et al., 2014), which was conducted between 2010 and 2014.^1^ The dataset included 90,350 respondents (mean age = 42.05 ± 16.48; 51.93% females) from 60 countries, with each country including over 1,000 respondents. However, as not all participants provided valid answers to the questions involved in this study, the number of participants varied across analyses.

### Measures

The descriptive information (i.e., mean, standard deviation, and reliability) of the variables under study by country is shown in the Supplementary Material.

#### Predictor

The search for meaning was measured by one item in the WVS that assessed respondents’ frequency of thinking about meaning and purpose of life (i.e., “How often, if at all, do you think about the meaning and purpose of life?”). I reverse keyed the item, with higher scores indicating greater effort to find meaning in life (4 = often, 1 = never). This item has been used in a previous research to capture the construct of the search for meaning, in which the results of Study 1 that used this single-item scale were consistent with Study 2 that measured the search for meaning with a 12-item scale (e.g., “At this moment, I am thinking more deeply about my life than I usually do”; Alter and Hershfield, 2014). In addition, I conducted a study using a convenience sample of 122 university students to investigate the relationship between this item and search for meaning in life. The results showed that thinking about meaning and purpose of life was moderately correlated with the search for meaning in life measured by the most widely used scale of search for meaning – the search for meaning subscale of the meaning in life questionnaire (MLQ; Steger et al., 2006) (*r* = 0.54, *p* < 0.001), but not correlated with the presence of meaning in life measured by the presence of meaning in life subscale of MLQ (*r* = 0.11, *p* = 0.24).^2^

#### Outcome Variables

I measured two categories of civic engagement: pro-environmental engagement and political engagement through self-report. Pro-environmental engagement was indexed by a sum of three items (internal consistency: α = 0.46). The first item measured individuals’ membership in an environmental organization (i.e., “could you tell me whether you are an active member, an inactive member or not a member of that type of organization?”). I dummy-coded the responses, with membership (active or inactive) coded as 1 and non-membership coded as 0. The second and third items measured whether the respondents “had given money to an environmental organization” and “participated in a demonstration for an environmental cause,” respectively, during the past 2 years. These items have been used in previous studies (e.g., Tam and Chan, 2018) to represent civic engagement in the environmental domain. Political engagement was indexed by a mean score of four items (internal consistency: α = 0.81). These items assessed respondents’ experience or likelihood of engagement in four political actions – signing a petition, joining in boycotts, attending peaceful demonstrations, and joining strikes. This scale has been used in previous studies (e.g., Alesina and Giuliano, 2011). I reverse keyed the items (3 = have done; 2 = might do; 1 = would never do), with higher scores indicating greater political engagement.

#### Moderators

At the individual level, I tested the moderating effects of both general values and domain-specific values. General values were indexed by the composite scores of openness to change (vs. conservation) and self-transcendence (vs. self-enhancement) based on 10 revised items of the Portrait Values Questionnaire (PVQ; Schwartz et al., 2001). This scale asked respondents to indicate the degree to which each description of 10 value types (i.e., power, achievement, hedonism, stimulation, universalism, benevolence, tradition, conformity, and security) described them. Following Schwartz (2012), I centered the response to each item within individuals in order to control for individual differences in acquiescent responding. The scale was reversed with higher scores indicating stronger endorsement of a certain value (5 = Very much like me; 1 = not at all like me). The openness scores were computed by subtracting the mean scores of tradition, conformity and security from the mean scores of self-direction, stimulation, and hedonism; the self-transcendence scores were computed by subtracting the mean scores of achievement and power from the mean scores of benevolence and universality (Schwartz et al., 2001). Higher scores indicate stronger endorsement of openness and self-transcendence values. A series of confirmatory factor analyses (CFAs) were performed to test the measurement invariance of the two scales: openness vs. conservation and self-transcendence vs. self-enhancement. I estimated one model with two latent factors: openness and conservation (OC model), and the other model with two latent factors: self-transcendence vs. self-enhancement (SS model). In the full sample model, the data fit the models well (OC model: RMSEA = 0.035, CFI = 0.989, TLI = 0.974; SS model: RMSEA = 0.038, CFI = 0.997, TLI = 0.984). In the multi-group models, the results yielded metric invariance (assuming the factor loadings to be equal across societies) with approximately good model fit (OC model: RMSEA = 0.080, CFI = 0.909, TLI = 0.863; SS model: RMSEA = 0.084, CFI = 0.954, TLI = 0.907). In other words, factor covariances and unstructured regression coefficients are comparable across societies.^3^ In the full sample, the openness vs. conservation scale had an alpha reliability of 0.61, and the self-transcendence vs. self-enhancement scale had an alpha reliability of 0.57, which was similar to the results of the fifth wave of WVS (Malka et al., 2014). The reliabilities of each specific society were presented in the Supplementary Material.

Additionally, the pro-environmental value was indexed by priority and personal importance. The single item about priority asked respondents to provide a weighting between environmental protection and economic growth (1 = “Protecting the environment should be given priority...”; 0 = “Economic growth and creating jobs should be the top priority…”). Personal importance was assessed by the item of the PVS that indicated universality (i.e., “Looking after the environment is important to this person…”). Finally, political values were indexed by interest and personal importance. Interest was assessed by a single item asking respondents to answer the question “How interested would you say you are in politics?” I reverse keyed the item, with higher scores indicating a stronger interest (1 = not at all interested; 4 = very interested). The personal importance of politics was measured by one item asking for a response to the question “How important is politics in your life?” I reverse keyed the item, with higher scores indicating greater importance (1 = not at all important; 4 = very important).

The society-level moderator, power distance, was derived from the index developed by Hofstede and associates (Hofstede, 1980; Hofstede et al., 2010). This is a societal-level variable that indicates the meaning system shared by residents in a certain society. Based on their original study of more than 116,000 employees at International Business Machine (IBM) in 40 countries (Hofstede, 1980) as well as other cross-cultural studies (Hofstede et al., 2010), they identified four dimensions to categorize the cultural differences across countries: power distance, individualism-collectivism, masculinity-femininity, and uncertainty avoidance. Hofstede (1980) argued that these cultural values represent societal characteristics rather than individual-level values orientations. I used the index of power distance in this study. Higher scores represent higher levels of power distance of society.

#### Control Variables

I also derived variables on respondents’ sex, age, education, and perceived income status from the WVS and controlled them in the analyses.

### Data Analysis Plan

To test the hypotheses, two series of multi-level analyses were conducted on the two outcome variables – pro-environmental engagement and political engagement. First, I established eight random-coefficient models that involved individual-level data only. These models were conducted to test the main effects of search for meaning and personal values as well as their interaction effect on civic engagement at the individual level. For each outcome for civic engagement, I tested four moderators separately, serving as a conceptual replication. All the variables at the individual level, except sex, were mean centered within each country. I also allowed the slope of the search for meaning to vary to examine whether there was any cross-national variation in the search for meaning-civic engagement association.

Next, I conducted eight slope-as-outcome models to test the moderating effect of power distance at the society level on the search for meaning-civic engagement association at the individual level. The scores of power distance at the society-level were standardized. An illustration of the model is shown below:

(1) $$\begin{array}{c} Individual\;level:\;CE_{ij}=\beta_{0j}+\beta_{1j}(search\;for\;meaning)+\beta_{2}(values)\\ +\beta_{3}(search\;for\;meaning\;\times \;values)+\beta_{4-7}(covariates)+r_{ij} \end{array}$$

(2) $$Society\;level\;:\;\beta_{0j}=\gamma_{00}+\gamma_{01}\;(power\;distance)+\mu_{0j}$$

(3) $$\beta_{1j}=\gamma_{10}+\gamma_{11}\;(power\;distance)+\mu_{1j}$$

In equation (1), CE*_ij_* is the civic engagement score for individual *i* in society *j*; β*_0j_* is the intercept for society *j*; β*_1j_* is the slope of search the for meaning for society *j*; β_2_ is the slope of values; β_3_ is the slope of the interaction between the search for meaning and values; β*_4-7_* denotes the slope of individual-level covariates; and *r_ij_* is the individual-level residual variance. In equation (2), γ_00_ is the grand intercept, denoting the grand mean of civic engagement; γ_01_ is the slope of power distance, denoting the main effect of power distance; and *u_0j_* is the residual society-level variance of civic engagement. In equation (3), γ_10_ is the grand slope, denoting the main effect of the search for meaning; γ_11_ is the slope of the society-level variable, representing the hypothesized cross-level interaction; and *u_1j_* is the residual society-level variance of the slope of the search for meaning.

## Results

Tables 1, 2 present the final results with both levels of predictors. The random-coefficient models revealed five important findings (see Supplementary Material, for more details). First, these models showed that the search for meaning was positively related to self-reported pro-environmental engagement beyond the effect of each personal value. Second, the interaction effect between the search for meaning and openness (vs. conservation) and that between the search for meaning and environmental priority were positive and significant on pro-environmental engagement. The simple slope analyses demonstrated that the positive relationship was stronger when people endorsed stronger values of openness (high openness: γ = 0.076, *SE* = 0.007; low openness: γ = 0.042, *SE* = 0.007; *p*s < 0.001) and prioritized environment wellness over economic growth (high priority: γ = 0.063, *SE* = 0.007; low priority: γ = 0.046, *SE* = 0.008; *p*s < 0.001). However, self-transcendence values and the importance of the environment did not moderate the associations between the search for meaning and pro-environmental engagement. To control the potential measurement variance across countries, I re-conducted the analyses by controlling the internal consistency of the scale of openness to change (vs. conservation) and the scale of self-transcendence (vs. self-enhancement) at societal level, respectively (for a similar practice, see Malka et al., 2014). The statistically significant interaction remained positive and significant after partialing out the internal consistency of the subscale of openness vs. conservation at societal level. The details of the results were presented in the Supplementary Material.

**Table 1:** Final models of pro-environmental engagement.

Outcome variable	Pro-environmental engagement			
	
Individual-level moderator	Openness vs. Conservation	Self-transcendence vs. Self-enhancement	Environmental priority	Importance of environment
*Level 1 – individual level*				
Intercept (γ_00_)	0.328 (0.029)^∗∗∗^	0.331 (0.030)^∗∗∗^	0.251 (0.029)^∗∗∗^	0.317 (0.029)^∗∗∗^
**SMIL (γ_10_)**	**0.059 (0.006**)^∗∗∗^	**0.056 (0.006**)^∗∗∗^	**0.046 (0.008)**^∗∗∗^	**0.045 (0.006)**^∗∗∗^
Values (β_2_)	0.044 (0.003)^∗∗∗^	0.038 (0.002)^∗∗∗^	0.116 (0.006)^∗∗∗^	0.070 (0.002)^∗∗∗^
**SMIL × Values (β_3_)**	**0.014 (0.003**)^∗∗∗^	**-0.003 (0.003)**	**0.018 (0.008)**^∗^	**0.005 (0.003)**
Gender (β_4_)	0.010 (0.006)	0.031 (0.006)^∗∗∗^	0.022 (0.006)^∗∗∗^	0.027 (0.006)^∗∗∗^
Age (β_5_)	0.002 (0.000)^∗∗∗^	0.000 (0.000)	0.001 (0.000)^∗∗∗^	0.000 (0.000)^∗^
Education (β_6_)	0.030 (0.002)^∗∗∗^	0.028 (0.002)^∗∗∗^	0.030 (0.002)^∗∗∗^	0.029 (0.001)^∗∗∗^
Income (β_7_)	0.013 (0.002)^∗∗∗^	0.016 (0.002)^∗∗∗^	0.014 (0.002)^∗∗∗^	0.017 (0.001)^∗∗∗^
*Level 2 – Societal level*				
Power distance (γ_01_)	-0.037 (0.029)	-0.037 (0.028)	-0.042 (0.029)	-0.038 (0.029)
*Cross-level interaction*				
**SMIL × Power distance (γ_11_)**	**-0.024 (.006)**^∗∗∗^	**-0.023 (0.006)**^∗∗∗^	**-0.029 (0.006)**^∗∗∗^	**-0.024 (0.006)**^∗∗∗^
*Residual variance (SE)*				
Level 1 variance (*r_ij_*)	0.405 (0.003)^∗∗∗^	0.417 (0.003)^∗∗∗^	0.402 (0.003)^∗∗∗^	0.392 (0.003)^∗∗∗^
Level 2 variance of intercept (*u_0j_*)	0.028 (0.007)^∗∗∗^	0.027 (0.007)^∗∗∗^	0.027 (0.007)^∗∗∗^	0.002 (0.001)^∗∗∗^
Level 2 variance of slope of search for meaning (*u_1j_*)	0.001 (0.000)^∗∗^	0.001 (0.000)^∗^	0.001 (0.000)^∗∗^	0.001 (0.000)^∗^
Number of respondents	44,791	42,004	43,118	47,313
Number of countries	34	32	33	34
*R*^2^ (level 2)^a^	6.39%	6.68%	8.65%	6.80%
Log likelihood	-43379.142	-41308.097	-41635.932	-45070.395
AIC	86786.28	82644.19	83299.86	90168.79
BIC	86908.22	82765.23	83421.27	90291.49


*Boldface highlights the results of major interest. SMIL = Search for meaning in life; ^*a*^*R^2^*(pseudo *R^2^*) shows the proportion of Level 2 variance that has been explained by the Level 2 predictors, by comparing each slope-as-outcome model with the random-coefficient model based on the same sample (Snijders and Bosker, 1999). ^∗^*p* < 0.05; ^∗∗^*p* < 0.01; ^∗∗∗^*p* < 0.001.*

**Table 2:** Final models of political engagement.

Outcome variable	Political Engagement			
	
Individual-level moderator	Openness vs. Conservation	Self-transcendence vs. Self-enhancement	Political Interest	Importance of Politics
*Level 1 – individual level*				
Intercept (*γ_00_*)	1.549 (0.028)^∗∗∗^	1.546 (0.028)^∗∗∗^	1.553 (0.028)^∗∗∗^	1.545 (0.028)^∗∗∗^
**SMIL (γ_10_)**	**0.052 (0.006)**^∗∗∗^	**0.049 (0.006)**^∗∗∗^	**0.036 (0.006)**^∗∗∗^	**0.045 (0.006)**^∗∗∗^
Values (*β_2_*)	0.037 (0.002)^∗∗∗^	0.024 (0.002)^∗∗∗^	0.141 (0.003)^∗∗∗^	0.089 (0.003)^∗∗∗^
**SMIL × Values (*β_3_*)**	**0.009 (0.002)**^∗∗∗^	**-0.002 (0.002)**	**0.012 (0.003)**^∗∗∗^	**0.021 (0.003)**^∗∗∗^
Gender (*β_4_*)	0.068 (0.005)^∗∗∗^	0.083 (0.005)^∗∗∗^	0.047 (0.005)^∗∗∗^	0.068 (0.005)^∗∗∗^
Age (*β_5_*)	-0.001 (0.000)^∗∗^	-0.001 (0.000)^∗∗∗^	-0.002 (0.000)^∗∗∗^	-0.002 (0.000)^∗∗∗^
Education (*β_6_*)	0.046 (0.001)^∗∗∗^	0.045 (0.001)^∗∗∗^	0.039 (0.001)^∗∗∗^	0.044 (0.001)^∗∗∗^
Income (*β_7_*)	0.001 (0.001)	0.004 (0.001)^∗∗^	0.000 (0.001)	0.002 (0.001)
*Level 2 – Societal level*				
Power distance (*γ_01_*)	-0.198 (0.027)^∗∗∗^	-0.194 (0.026)^∗∗∗^	-0.196 (0.027)^∗∗∗^	-0.197 (.027)^∗∗∗^
*Cross-level interaction*				
**SMIL x Power distance (*γ_11_*)**	**-0.014 (0.006)**^∗^	**-0.013 (0.006)**^∗^	**-0.013 (0.006)**^∗^	**-0.013 (**0**.006)**^∗^
*Residual variance (SE)*				
Level 1 variance (*r_ij_*)	0.001 (0.001)^∗∗∗^	0.001 (0.001)^∗∗∗^	0.224 (0.002)^∗∗∗^	0.234 (0.002)^∗∗∗^
Level 2 variance of intercept (*u_0j_*)	0.024 (0.006) ^∗∗∗^	0.023 (0.006) ^∗∗∗^	0.025 (0.006)^∗∗∗^	0.025 (0.006)^∗∗∗^
Level 2 variance of slope of search for meaning (*u_1j_*)	0.001 (0.000)^∗∗^	0.001 (0.000)^∗∗^	0.001 (0.000)^∗∗∗^	0.001 (0.000)^∗∗^
Number of respondents	41,232	38,560	43,848	43,501
Number of countries	33	31	33	33
*R*^2^ (level 2)^a^	23.07%	62.75%	60.64%	60.83%
Log likelihood	-28940.283	-26936.146	-29539.681	-30220.1
AIC	57908.57	53900.29	59107.36	60468.2
BIC	58029.34	54020.13	59229	60589.73


*Boldface highlights the results of major interest. SMIL = Search for meaning in life; ^*a*^*R^2^* (pseudo*R^2^*) shows the proportion of Level 2 variance that has been explained by the Level 2 predictors, by comparing each slope-as-outcome model with the random-coefficient model based on the same sample (Snijders and Bosker, 1999). ^∗^*p* < 0.05; ^∗∗^*p* < 0.01; ^∗∗∗^*p* < 0.001.*

Third, the search for meaning was positively related to self-reported political engagement beyond the effect of each personal value. Fourth, except self-transcendence values, personal values significantly moderated the relationship between the search for meaning and political engagement. The simple slope analyses showed that when people endorsed stronger values of openness (vs. conservation) (γ = 0.063, *SE* = 0.007; γ = 0.042, *SE* = 0.007; *p*s < 0.001), had stronger interests in politics (high interest: γ = 0.047, *SE* = 0.006; low interest: γ = 0.024, *SE* = 0.007; *p*s < 0.001) or attributed greater importance to politics (high importance: γ = 0.065, *SE* = 0.007; low importance: γ = 0.024, *SE* = 0.007; *p*s < 0.001), the positive relationship between the search for meaning and political engagement was stronger. The statistically significant interaction remained positive and significant after excluding the internal consistency of the subscale of openness vs. conservation at societal level. Finally, as expected, the random effects of slope were significant in the models of pro-environmental engagement and the models of political engagement, which suggested that the search for meaning-civic engagement associations varied across societies.

I then added the society-level moderator, power distance, into the slope-as-outcome models. The results of individual-level predictors remained consistent with the random-coefficient models. As expected, the cross-level interaction between the search for meaning and power distance was negative and significant in all models. To illustrate, in the model of pro-environmental engagement with openness (vs. conservation) as personal values, the association between the search for meaning and pro-environmental engagement was weaker in a society with higher power distance (γ = 0.035, *SE* = 0.009, *p* < 0.001) relative to that with lower power distance (γ = 0.083, *SE* = 0.008, *p* < 0.001). Similarly, in the model of political engagement with openness (vs. conservation) as personal values, the association between the search for meaning and political engagement was weaker in a society with higher power distance (γ = 0.066, *SE* = 0.008, *p* < 0.001) relative to that with lower power distance (γ = 0.038, *SE* = 0.009, *p* < 0.001). Other models showed similar results. The results of the simple slope effects of all models are presented in online Supplementary Material.^4^

## Discussion

With reference to meaning regulation theories (e.g., van Tilburg and Igou, 2011), this study formulated and obtained initial support for a proposition that explains the individual differences in the associations between the search for meaning in terms of thinking about meaning and purpose of life and civic engagement from the perspective of values. Specifically, people who more strongly engaged in the reflection on purpose and meaning reported higher engagement in pro-environmental and political civic activities. These associations were stronger when people held stronger values of openness to change (vs. conservation), attributed greater importance to the particular domain of civic issues, or lived in a society with a lower power distance. As the first study that probes the behavioral outcomes of the search for meaning as a function of values, it makes two significant contributions to the understanding of people’s spiritual endeavor on meaning search.

In line with previous research that captures the civic intention of people who are searching for meaning (Scales et al., 2014; van Tilburg and Igou, 2017), this study reaffirms that when people are pondering over big questions about meaning and purpose, they may attempt to discover what gives them meaning by engaging in pro-environmental activities or taking up political causes. Civic engagement may provide people with an answer, though inconclusive, about where they belong, how much self-worth they possess, the extent to which they can control and predict their lives, and whether their lives can become immortal. All of these gains consolidate the building blocks of meaning in life. This study advances the theoretical understanding by examining who are more likely to search for meaning through civic engagement. Previous studies (e.g., Brassai et al., 2012) have found other behavioral correlates of the search for meaning, including less aggressive behavior, less irresponsible academic behavior and more healthy eating behavior among adolescents. The current study goes beyond these findings by suggesting that people may engage in certain meaningful behaviors based on their personal values and cultural values.

It is well established that values serve as a guide for human behavior (Roccas and Sagiv, 2010). Civic engagement usually requires people to step out of their comfort zone, such as by meeting new people, voicing their opinions, and making changes in the community and society. People who are open to change probably regard such personal or social changes to be worthwhile and desirable and thus are more likely to search for meaning through civic engagement. Additionally, individuals’ specific values in the civic domain may influence their behavioral choices. When people prioritize environmental protection over economic growth, they perceive engagement in pro-environmental actions to be a more desirable approach to saving the earth and thus more meaningful. When people have a stronger interest in or attribute greater importance to politics, they consider taking political actions to be desirable and meaningful.

However, self-transcendence values and environmental values do not intervene in the associations between the search for meaning and civic engagement. The perceived meaningfulness of civic engagement may not depend on these values. For instance, self-transcendence values do not make people perceive civic engagement to be more meaningful. Individuals may consider other values of civic engagement (e.g., strengthening social connections, obtaining self-development, and gaining career-related experience; Clary et al., 1998) when they judge its meaningfulness. Alternatively, people with stronger self-transcendence values and environmental values are motivated to engage in civic activities regardless of their motivation to quest meaning. In other words, perceived meaningfulness does not affect these people’s behavioral selection. Future studies should examine these two possibilities by measuring the perceived meaningfulness of civic engagement directly.

Another notable contribution of this study is that it advances our understanding of behavioral outcomes (though based on self-report) in with reference to the cultural context in which people operate. Personal values and cultural values are differentiated to the extent that they can work independently and interactively in shaping individuals’ behaviors (Roccas and Sagiv, 2010). The meaning of a goal varies across contexts, which may affect how likely people are to engage in specific behavior to serve this goal. Steger et al. (2008b) suggest that the psychological correlates of the search for meaning might vary as a function of cultural values; however, the authors tested cultural variations by relying on a country proxy without directly probing cultural values. The current study provides initial evidence by finding that the associations between the search for meaning and civic engagement vary according to the power distance of a society. When a society upholds (vs. downplays) equality in power, members of the society are more likely to attach importance to individual contributions to solving public issues or improving the common good; thus, they are more likely to feel encouraged to reach their life meaning by pursuing these goals. These findings echo the argument that power distance deters people’s contribution to the welfare of others and society (e.g., charity behavior; Winterich and Zhang, 2014), and highlight the importance of cultural context in individuals’ meaning quest. Altogether, the current findings suggest how people might select meaningful behavior among multiple options when they want to reach meaningfulness, and future research can work to further elucidate this behavioral selection process.

Several caveats should be noted regarding this study. The first limitation concerns measurement used in this study, including single-item problem and measurement equivalence across countries of the items using Likert scale. Most of the predictors were assessed by single item. For example, the search for meaning was narrowly operationalized as thinking about the meaning and purpose of life. I thus encourage future studies to validate this hypothesized model with more comprehensive scales (e.g., The Meaning in Life Questionnaire; Steger et al., 2006). Recent research has identified two dimensions of the search for meaning in life (i.e., life reflection and perceived value of meaning; Kim et al., 2018). Thinking about meaning and purpose of life is one of life reflection acts, while future studies might extend the current research by investigating how the other dimension is related to civic engagement. Moreover, personal values orientations were assessed by the briefest version of PVQ, though comprising multiple items, and thus it would be better if future studies could adopt a version of PVQ (e.g., 21-item version) with wider coverage of values and evidence for measurement invariance across countries (e.g., Davidov et al., 2008; Heim et al., 2017). Besides, it cannot exclude the cross-cultural variance in response style that may jeopardize measurement invariance. WVS research team assumed response style variation to be a component of the values and behaviors under investigation (Smith et al., 2016) and thus did not attempt to eliminate it. Future cross-national studies would benefit from a more sophisticated design to control response style variances.

Second, the current study was based on a correlational design; thus, causal relationships among variables could not be confirmed. The reverse path (civic engagement → search for meaning) might be plausible, but it is not clear whether or how civic engagement changes one’s desires and practices in the quest for meaning in the long run (i.e., enhancing or reducing the search). Similarly, it is theoretically sound that values guide one’s behavior but the reverse direction is possible at both individual- and society-levels (cf. Rudnev and Vauclair, 2018). Future research would benefit from using a longitudinal design to disentangle the relationships between variables that may be reciprocal over time. Additionally, it is possible that a third variable accounts for the association between the search for meaning and civic engagement. For example, people who like thinking about meaning and those engaging in civic actions may share similar personality characteristics such as stronger curiosity (Steger et al., 2008a; Lechner et al., 2018). Future study can use an experimental design that manipulates people’s desire of search for meaning (see an example, van Tilburg and Igou, 2017) to exclude such a possibility. Finally, the number of countries involved in the society-level analyses may not have been sufficient to represent cultural heterogeneity, and thus, the influence of power distance might be underestimated. I look forward to further validation by including more societies.

## Ethics Statement

No ethics approval was required as the present study did not involve new data collection. All the analyses were based on existing public archival data.

## Author Contributions

The author confirms being the sole contributor of this work and has approved it for publication.

## Conflict of Interest Statement

The author declares that the research was conducted in the absence of any commercial or financial relationships that could be construed as a potential conflict of interest.

## Footnotes

1 The sixth wave of WVS is the only one wave of assessment that investigated individuals’ self-reported pro-environmental behavior.

2 The sample consists of students who were requested to participate in a psychology research to fulfill the credit requirement of an introductory psychology course (*N* = 122, Mean age = 21.5, *SD* = 1.37, female = 41.8%). Particular caution is still warranted in generalizing the current findings.

3 It is noted that some technical problems occurred in the estimation of configural invariance (assuming equal latent constructs across societies) prior to the estimation of metric invariance, which rendered the models of configural invariance failing to converge. Specifically, the standardized errors could not be computed. Caution is still warranted for the use of 10-item PVQ given the restricted number of items that pose a great challenge to the measurement invariance test (see Morselli et al., 2012).

4 The online supplementary document also presents Supplementary Analyses ruling out the influence of gross domestic product (GDP).

## References

[B1] Albanesi C. Cicognani E. Zani B. (2007). Sense of community, civic engagement and social well-being in Italian adolescents. *J. Commun. Appl. Soc. Psychol.* 17 387–406. 10.1002/casp.903

[B2] Alesina A. Giuliano P. (2011). Family ties and political participation. *J. Eur. Econ. Assoc.* 9 817–839. 10.1111/j.1542-4774.2011.01024.x

[B3] Alter A. L. Hershfield H. E. (2014). People search for meaning when they approach a new decade in chronological age. *Proc. Natl. Acad. Sci. U.S.A.* 111 17066–17070. 10.1073/pnas.1415086111 25404347 PMC4260584

[B4] Benson P. L. Scales P. C. Syvertsen A. K. Roehlkepartain E. C. (2012). Is youth spiritual development a universal developmental process? An international exploration. *J. Posit. Psychol.* 7 453–470. 10.1080/17439760.2012.732102

[B5] Brassai L. Piko B. F. Steger M. F. (2012). Existential attitudes and eastern european adolescents’ problem and health behaviors: highlighting the role of the search for meaning in life. *Psychol. Rec.* 62 719–734. 10.1007/bf03395831

[B6] Brooks G. M. (ed) (2017). *Civic Engagement: Perspectives, Roles, and Impacts*. New York, NY: Nova Science Publishers.

[B7] Brown K. M. Hoye R. Nicholson M. (2012). Self-esteem, self-efficacy, and social connectedness as mediators of the relationship between volunteering and well-being. *J. Soc. Serv. Res.* 38 468–483. 10.1080/01488376.2012.687706

[B8] Chan H. W. Pong V. Tam K. P. (2019). Cross-national variation of gender differences in environmental concern: testing the sociocultural hindrance hypothesis. *Environ. Behav.* 51 81–108. 10.1177/0013916517735149

[B9] Clary E. G. Snyder M. Ridge R. D. Copeland J. Stukas A. A. Haugen J. (1998). Understanding and assessing the motivations of volunteers: a functional approach. *J. Pers. Soc. Psychol.* 74 1516–1530. 10.1037//0022-3514.74.6.1516 9654757

[B10] Cohen E. H. Valencia J. (2008). Political protest and power distance. towards a typology of political participation. *Bull. Soc. Methodol.* 99 54–72. 10.1177/075910630809900105

[B11] Davidov E. Schmidt P. Schwartz S. H. (2008). Bringing values back in: the adequacy of the european social survey to measure values in 20 countries. *Public Opin. Q.* 72 420–445. 10.1093/poq/nfn035

[B12] Frankl V. (1959). *Man’s Search for Meaning*. New York, NY: Washington Square.

[B13] Greenaway K. H. Haslam S. A. Cruwys T. Branscombe N. R. Ysseldyk R. Heldreth C. (2015). From “we” to “me”: group identification enhances perceived personal control with consequences for health and well-being. *J. Pers. Soc. Psychol.* 109 53–74. 10.1037/pspi0000019 25938701

[B14] Greenberg J. Kosloff S. (2008). Terror management theory: implications for understanding prejudice, stereotyping, intergroup conflict, and political attitudes. *Soc. Pers. Psychol. Compass.* 2 1881–1894. 10.1111/j.1751-9004.2008.00144.x

[B15] Heim E. Scholten S. Maercker A. Xiu D. Cai D. Gao Z. H. (2017). Students’ value orientations in contemporary China: analysis of measurement invariance and latent mean differences in comparison with students from Germany and Russia. *J. Cross-Cult. Psychol.* 48 511–531. 10.1177/0022022117696800

[B16] Heine S. J. Proulx T. Vohs K. D. (2006). The meaning maintenance model: on the coherence of social motivations. *Pers. Soc. Psychol. Rev.* 10 88–110. 10.1207/s15327957pspr1002_1 16768649

[B17] Hofstede G. (1980). *Culture’s Consequences: International Differences in Work Related Values*. Beverly Hills, CA: SAGE.

[B18] Hofstede G. Hofstede G. J. Minkov M. (2010). *Culture and Organization: Software for the Mind*, 3rd Edn. New York, NY: McGraw-Hill.

[B19] Inglehart R. C. Haerpfer A. Moreno C. Welzel K. Kizilova J. Diez-Medrano M. (eds) (2014)). *World Values Survey: Round Six - Country-Pooled Datafile Version*. Madrid: JD Systems Institute.

[B20] Kim J. Schlegel R. J. Seto E. Hicks J. A. (2018). Thinking about a new decade in life increases personal self-reflection: a replication and reinterpretation of Alter and Hershfield’s (2014) findings. *J. Pers. Soc. Psychol.* [Epub ahead of print] 29952577 10.1037/pspp0000199

[B21] Lechner C. M. Pavlova M. K. Sortheix F. M. Silbereisen R. K. Salmela-Aro K. (2018). Unpacking the link between family socioeconomic status and civic engagement during the transition to adulthood: do work values play a role? *Appl. Dev. Sci.* 22 270–283. 10.1080/10888691.2017.1291352

[B22] Malka A. Soto C. J. Inzlicht M. Lelkes Y. (2014). Do needs for security and certainty predict cultural and economic conservatism? A cross-national analysis. *J. Pers. Soc. Psychol.* 106 1031–1051. 10.1037/a0036170 24841103

[B23] Martela F. Ryan R. M. (2016). Prosocial behavior increases well-being and vitality even without contact with the beneficiary: causal and behavioral evidence. *Motiv. Emot.* 40 351–357. 10.1007/s11031-016-9552-z

[B24] Morselli D. Spini D. Devos T. (2012). Human values and trust in institutions across countries: a multilevel test of Schwartz’s hypothesis of structural equivalence. *Surv. Res. Methods* 6 49–60.

[B25] Ohmer M. L. (2007). Citizen participation in neighborhood organizations and its relationship to volunteers’ self-and collective efficacy and sense of community. *Soc. Work Res.* 31 109–120. 10.1093/swr/31.2.109

[B26] Pancer S. M. Pratt M. Hunsberger B. Alisat S. (2007). Community and political involvement in adolescence: what distinguishes the activists from the uninvolved? *J. Commun. Psychol.* 35 741–759. 10.1002/jcop.20176

[B27] Roccas S. Sagiv L. (2010). Personal values and behavior: taking the cultural context into account. *Soc. Pers. Psychol. Compass.* 4 30–41. 10.1111/j.1751-9004.2009.00234.x

[B28] Rohan M. J. (2000). A rose by any name? The values construct. *Pers. Soc. Psychol. Rev.* 4 255–277. 10.1207/S15327957PSPR0403_4 29116982

[B29] Rudnev M. Vauclair C. M. (2018). The link between personal values and frequency of drinking depends on cultural values: a cross-level interaction approach. *Front. Psychol.* 9:1379. 10.3389/fpsyg.2018.01379 30131741 PMC6090463

[B30] Scales P. C. Syvertsen A. K. Benson P. L. Roehlkepartain E. C. Sesma A. Jr. (2014). “Relation of spiritual development to youth health and well-being: evidence from a global study,” in *Handbook of Child Well-Being*, eds Ben-Arieh A. Casa F. Frones I. Korbin J. (Dordrecht: Springer), 1101–1135. 10.1007/978-90-481-9063-8_41

[B31] Schwartz S. (2006). A theory of cultural value orientations: explication and applications. *Comp. Socio.* 5 137–182. 10.1163/156913306778667357

[B32] Schwartz S. H. (1999). A theory of cultural values and some implications for work. *Appl. Psychol.* 48 23–47. 10.1080/026999499377655

[B33] Schwartz S. H. (2012). An overview of the Schwartz theory of basic values. *Online Read. Psychol. Cult.* 2:11 10.9707/2307-0919.1116

[B34] Schwartz S. H. Melech G. Lehmann A. Burgess S. Harris M. Owens V. (2001). Extending the cross-cultural validity of the theory of basic human values with a different method of measurement. *J. Cross Cult. Psychol.* 32 519–542. 10.1177/0022022101032005001

[B35] Smith P. B. Vignoles V. L. Becker M. Owe E. Easterbrook M. J. Brown R. (2016). Individual and culture-level components of survey response styles: a multi-level analysis using cultural models of selfhood. *Int. J. Psychol.* 51 453–463. 10.1002/ijop.12293 27374874

[B36] Snijders T. A. B. Bosker R. J. (1999). *Multilevel analysis: An introduction to basic and advanced multilevel modeling*. Thousand Oaks, CA: SAGE.

[B37] Stavrova O. Luhmann M. (2016). Social connectedness as a source and consequence of meaning in life. *J. Posit. Psychol.* 11 470–479. 10.1080/17439760.2015.1117127

[B38] Steger M. F. Frazier P. Oishi S. Kaler M. (2006). The meaning in life questionnaire: assessing the presence of and search for meaning in life. *J. Couns. Psychol.* 53 80–93. 10.1080/00223891.2013.765882 23406365

[B39] Steger M. F. Kashdan T. B. Sullivan B. A. Lorentz D. (2008a). Understanding the search for meaning in life: personality, cognitive style, and the dynamic between seeking and experiencing meaning. *J. Pers.* 76 199–228. 10.1111/j.1467-6494.2007.00484.x 18331281

[B40] Steger M. F. Kawabata Y. Shimai S. Otake K. (2008b). The meaningful life in Japan and the United States: levels and correlates of meaning in life. *J. Res. Pers.* 42 660–678. 10.1016/j.jrp.2007.09.003

[B41] Talò C. Mannarini T. Rochira A. (2014). Sense of community and community participation: a meta-analytic review. *Soc. Indic. Res.* 117 1–28. 10.1007/s11205-013-0347-2

[B42] Tam K. P. Chan H. W. (2018). Generalized trust narrows the gap between environmental concern and pro-environmental behavior: multilevel evidence. *Glob. Environ. Change* 48 182–194. 10.1016/j.gloenvcha.2017.12.001

[B43] van Tilburg W. A. Igou E. R. (2011). On boredom and social identity: a pragmatic meaning-regulation approach. *Pers. Soc. Psychol. Bull.* 37 1679–1691. 10.1177/0146167211418530 21844095

[B44] van Tilburg W. A. Igou E. R. (2013). On the meaningfulness of behaviour: an expectancy x value approach. *Motiv. Emot.* 37 373–388. 10.1007/s11031-012-9316-3

[B45] van Tilburg W. A. Igou E. R. (2017). Can boredom help? Increased prosocial intentions in response to boredom. *Self Ident.* 16 82–96. 10.1080/15298868.2016.1218925

[B46] van Tongeren D. R. Green J. D. Davis D. E. Hook J. N. Hulsey T. L. (2016). Prosociality enhances meaning in life. *J. Posit. Psychol.* 11 225–236. 10.1080/17439760.2015.1048814

[B47] Winterich K. P. Zhang Y. (2014). Accepting inequality deters responsibility: how power distance decreases charitable behavior. *J. Consum. Res.* 41 274–293. 10.1086/675927

[B48] Zimmerman M. A. Rappaport J. (1988). Citizen participation, perceived control, and psychological empowerment. *Am. J. Commun. Psychol.* 16 725–750. 10.1007/bf00930023 3218639

