# Supplementary material for: Is Searching for Meaning in Life Related to Civic Engagement?: Individual- and Society-Level Moderators
Source: Front Psychol. 2019 Jun 26;10:1334. doi: 10.3389/fpsyg.2019.01334 (PMC6607855; doi:10.3389/fpsyg.2019.01334)
Supplement: Supplementary file 1 [file Table_1.DOCX]

Supplementary Material

This supplementary document comprises two parts. The first part reports the descriptive information of the variables under investigation by country. Table S1 presents the mean scores, standard deviation as well as internal consistencies. Table S2 presents the correlations between the search for meaning and civic engagement. The second part shows the detailed statistic results. Table S3a and S3b present the results of individual level analyses (i.e., random coefficient model). Table S4a and S4b present the results of simple slope effects. Table S5a and S5b present the results of supplementary analyses that controlled economic development index of each society.

**Part 1**

Table S1. Descriptive information of study variables

| Variables | | SMIL | | Environmental Engagement | | Political Engagement | | Openness for change vs. Conservation | | Self-transcendence vs. Self-enhancement | | Environmental Priority | Importance of Environment | Political Interest | Importance of Politics | |
| --- | --- | --- | --- | --- | --- | --- | --- | --- | --- | --- | --- | --- | --- | --- | --- | --- |
| Country/Area | | Mean (SD) | | Mean(SD) | α | Mean(SD) | α | Mean(SD) | α | Mean(SD) | α | Mean(SD) | Mean(SD) | Mean(SD) | Mean(SD) | |
| Algeria | | 2.97 (1.03) | | .15 (.42) | .35 | 1.53 (.60) | .89 | -.63 (1.47) | .46 | .12 (1.63) | .53 | .42 (.49) | 4.38 (1.48) | 2.28 (1.15) | 2.16 (.99) | |
| Azerbaijan | | 3.30 (.75) | | .08 (.34) | .42 | 1.09 (.27) | .76 | -1.12 (1.20) | .49 | .60 (1.35) | .55 | .36 (.48) | 4.16 (1.39) | 2.07 (.91) | 2.07 (.89) | |
| Argentina | | 2.71 (.98) | | .28 (.61) | .46 | 1.63 (.52) | .76 | .00 (1.34) | .44 | 1.19 (1.33) | .55 | .64 (.48) | 4.31 (1.18) | 2.12 (.95) | 2.14 (.95) | |
| Australia | | 3.14 (.83) | | .51 (.79) | .51 | 2.06 (.51) | .75 | -.76 (1.41) | .39 | 1.55 (1.39) | .46 | .61 (.49) | 4.39 (1.23) | 2.52 (.81) | 2.63 (.94) | |
| Bahrain | | 2.88 (.84) | | - | - | - | - | -.07 (.93) | .49 | .04 (1.07) | .46 | .48 (.50) | 4.29 (1.31) | 2.83 (.89) | 2.84 (.99) | |
| Armenia | | 3.60 (.70) | | .07 (.30) | .41 | 1.22 (.40) | .79 | -1.63 (1.35) | .41 | 1.15 (1.31) | .44 | .45 (.50) | 4.29 (1.21) | 2.09 (.98) | 2.06 (.96) | |
| Brazil | | 3.30 (.87) | | .19 (.50) | .43 | 1.70 (.51) | .69 | -.91 (1.31) | .37 | 1.96 (1.29) | .23 | .67 (.47) | 4.90 (1.00) | 2.25 (1.01) | 2.03 (1.02) | |
| Belarus | | 3.09 (.90) | | .10 (.34) | .23 | - | - | -.95 (1.60) | .58 | .58 (1.63) | .48 | .60 (.49) | 4.02 (1.24) | 2.18 (.85) | 2.29 (.84) | |
| Chile | | 3.07 (.80) | | .41 (.71) | .46 | 1.54 (.58) | .80 | -.42 (1.28) | .56 | 1.15 (1.48) | .53 | .73 (.45) | 4.43 (1.31) | 1.94 (.90) | 1.91 (.91) | |
| China | | 2.53 (.84) | | .08 (.31) | .32 | 1.38 (.46) | .89 | -.90 (1.32) | .43 | .52 (1.17) | .66 | .68 (.47) | 4.17 (1.15) | 2.46 (.80) | 2.37 (.94) | |
| Taiwan | | 2.99 (.88) | | .46 (.67) | .31 | 1.35 (.44) | .75 | -.93 (1.28) | .38 | 1.63 (1.32) | .47 | .63 (.48) | 4.32 (1.11) | 2.20 (.84) | 2.00 (.84) | |
| Colombia | | 3.40 (.84) | | .44 (.71) | .39 | 1.65 (.56) | .79 | -.92 (1.32) | .39 | 1.67 (1.33) | .37 | .68 (.47) | 5.26 (1.02) | 1.98 (.95) | 1.81 (.94) | |
| Cyprus | | 3.38 (.74) | | .36 (.69) | .51 | 1.73 (.59) | .82 | -.93 (1.42) | .43 | 1.29 (1.41) | .28 | .48 (.50) | 4.82 (1.23) | 2.27 (1.02) | 2.26 (.98) | |
| Ecuador | | 3.32 (.74) | | .18 (.49) | .46 | 1.27 (.41) | .75 | -.42 (1.00) | .64 | 1.05 (1.32) | .44 | .63 (.48) | 4.76 (1.24) | 2.45 (1.07) | 2.09 (.96) | |
| Estonia | | 3.05 (.83) | | .11 (.37) | .37 | 1.44 (.50) | .82 | -.83 (1.39) | .48 | 1.05 (1.38) | .45 | .52 (.50) | 4.15 (1.18) | 2.12 (.82) | 2.27 (.84) | |
| Variables | | SMIL | | Environmental Engagement | | Political Engagement | | Openness for change vs. Conservation | | Self-transcendence vs. Self-enhancement | | Environmental Priority | Importance of Environment | Political Interest | Importance of Politics | |
| Country/Area | | Mean (SD) | | Mean(SD) | α | Mean(SD) | α | Mean(SD) | α | Mean(SD) | α | Mean(SD) | Mean(SD) | Mean(SD) | Mean(SD) | |
| Georgia | 3.71 (.56) | | .12 (.38) | | .25 | 1.33 (.48) | .76 | -1.69 (1.33) | .41 | 1.36 (1.19) | .53 | .61 (.49) | 5.12 (.88) | 2.27 (.99) | 2.16 (.93) |  |
| Palestine | 3.15 (.86) | | .18 (.46) | | .28 | 1.59 (.60) | .79 | -.78 (1.17) | .61 | .56 (1.11) | .69 | .50 (.50) | 4.49 (1.21) | 2.49 (.98) | 2.60 (.88) |  |
| Germany | 2.90 (.91) | | .28 (.59) | | .45 | 1.88 (.55) | .80 | -.29 (1.49) | .48 | .42 (1.44) | .35 | .53 (.50) | 4.07 (1.25) | 2.41 (.83) | 2.74 (.91) |  |
| Ghana | 3.39 (.74) | | .28 (.55) | | .29 | 1.33 (.41) | .79 | -.90 (1.08) | .30 | .27 (1.06) | .51 | .50 (.50) | 5.05 (.98) | 2.39 (1.08) | 2.36(1.07) |  |
| Hong Kong | 3.09 (.75) | | .71 (.80) | | .35 | 1.71 (.54) | .77 | -.49 (1.07) | .50 | .81 (1.22) | .53 | .61 (.49) | 4.36 (1.13) | 2.47 (.77) | 2.22 (.82) |  |
| India | 2.86 (.95) | | .64 (.87) | | .53 | 1.67 (.60) | .77 | -.47 (1.29) | .58 | .58 (1.59) | .53 | .62 (.49) | 4.34 (1.64) | 2.42 (1.06) | 2.29(1.02) |  |
| Iraq | 3.02 (.87) | | .09 (.35) | | .37 | 1.40 (.49) | .72 | -.86 (1.14) | .34 | .59 (1.20) | .46 | .47 (.50) | 4.72 (1.39) | 2.27 (1.00) | 2.37 (.89) |  |
| Japan | 3.04 (.65) | | .16 (.40) | | .21 | 1.56 (.47) | .81 | -.41 (.99) | .40 | .93 (1.07) | .59 | .43 (.50) | 3.67 (1.06) | 2.94 (.79) | 2.76 (.75) |  |
| Kazakhstan | 2.87 (.83) | | .13 (.45) | | .56 | 1.17 (.32) | .81 | -1.03 (1.34) | .22 | .53 (1.42) | .47 | .56 (.50) | 3.97 (1.25) | 2.41 (.87) | 2.37 (.84) |  |
| Jordan | 3.38 (.80) | | .16 (.43) | | .27 | 1.16 (.36) | .67 | -.93 (1.14) | .49 | .63 (1.21) | .54 | .37 (.48) | 5.09 (1.11) | 2.20 (1.07) | 2.14 (.96) |  |
| South Korea | 3.28 (.70) | | .31 (.62) | | .43 | 1.74 (.51) | .81 | -.25 (1.21) | .56 | .56 (1.26) | .53 | .56 (.50) | 4.06 (1.26) | 2.63 (.82) | 2.32 (.82) |  |
| Kuwait | 3.14 (.89) | | .36 (.69) | | .55 | - | - | -.69 (1.26) | .48 | .33 (1.25) | .52 | .33 (.47) | 4.60 (1.35) | 2.80 (1.07) | 2.70 (.93) |  |
| Kyrgyzstan | 3.52 (.70) | | .34 (.65) | | .43 | 1.20 (.36) | .84 | -.43 (1.34) | .55 | .38 (1.40) | .46 | .61 (.49) | 4.30 (1.39) | 2.58 (.84) | 2.79 (.91) |  |
| Lebanon | 2.87 (.93) | | .48 (.76) | | .42 | 1.53 (.53) | .67 | -.25 (1.15) | .52 | .30 (1.35) | .39 | .44 (.50) | 4.44 (1.38) | 2.46 (1.09) | 2.51 (.95) |  |
| Libya | 3.38 (.83) | | .27 (.57) | | .46 | 1.40 (.48) | .81 | -1.16 (1.26) | .42 | 1.20 (1.33) | .44 | .60 (.49) | 5.09 (1.18) | 2.72 (1.08) | 2.66(1.02) |  |
| Malaysia | 3.45 (.68) | | .37 (.67) | | .46 | 1.12 (.31) | .81 | -1.25 (1.33) | .33 | .71 (1.38) | .48 | .77 (.42) | 4.71 (1.11) | 2.60 (.90) | 2.40 (.80) |  |
| Variables | | SMIL | | Environmental Engagement | | Political Engagement | | Openness for change vs. Conservation | | Self-transcendence vs. Self-enhancement | | Environmental Priority | Importance of Environment | Political Interest | Importance of Politics | |
| Country/Area | | Mean (SD) | | Mean(SD) | α | Mean (SD) | α | Mean(SD) | α | Mean(SD) | α | Mean(SD) | Mean(SD) | Mean(SD) | Mean(SD) | |
| Mexico | 3.29 (.85) | | .45 (.71) | | .40 | 1.49 (.44) | .67 | -.82 (1.27) | .41 | 1.68 (1.39) | .30 | .65 (.48) | 4.98 (1.16) | 2.39 (1.02) | 2.05 (.93) |  |
| Morocco | 3.47 (.78) | | .08 (.36) | | .44 | 1.22 (.42) | .81 | -1.22 (1.48) | .47 | - | .34 | .72 (.45) | 4.53 (1.33) | 1.65 (.92) | 1.64 (.82) |  |
| Netherlands | 2.90 (.76) | | .45 (.70) | | .37 | 1.81 (.48) | .74 | -.28 (1.26) | .45 | 1.55 (1.37) | .45 | .45 (.50) | 3.90 (1.20) | 2.31 (.73) | 2.70 (.87) |  |
| New Zealand | 3.12 (.89) | | .50 (.78) | | .62 | 2.11 (.52) | .75 | -.48 (1.33) | .44 | 1.41 (1.36) | .46 | .52 (.50) | 4.25 (1.22) | 2.45 (.79) | 2.65 (.85) |  |
| Nigeria | 3.46 (.65) | | .54 (.80) | | .48 | 1.62 (.53) | .73 | -.36 (.98) | .55 | .10 (1.02) | .67 | .35 (.48) | 4.79 (1.13) | 2.48 (1.02) | 2.56(1.04) |  |
| Pakistan | 3.25 (.89) | | .33 (.69) | | .52 | 1.50 (.57) | .79 | -.16 (.87) | .74 | .20 (1.13) | .60 | .47 (.50) | 4.36 (1.31) | 2.20 (.98) | 2.44 (.98) |  |
| Peru | 3.11 (.92) | | .38 (.68) | | .48 | 1.57 (.47) | .70 | -.82 (1.14) | .57 | 1.26 (1.22) | .63 | .69 (.46) | 4.55 (1.19) | 2.26 (.95) | 1.98 (.91) |  |
| Philippines | 3.48 (.66) | | .50 (.73) | | .58 | 1.26 (.39) | .68 | -.78 (1.06) | .51 | 1.16 (1.24) | .50 | .65 (.48) | 4.93 (1.07) | 2.71 (1.00) | 2.63 (.95) |  |
| Poland | 2.89 (.88) | | .12 (.37) | | .46 | 1.59 (.49) | .73 | -.90 (1.23) | .28 | 1.19 (1.18) | .39 | .42 (.49) | 4.80 (.97) | 2.17 (.83) | 2.26 (.89) |  |
| Qatar | 3.53 (.77) | | .41 (.72) | | .39 | - | - | -1.09 (1.00) | .39 | .99 (1.01) | .39 | .64 (.48) | 5.50 (.78) | 2.84 (.98) | 2.71 (.87) |  |
| Romania | 3.35 (.83) | | .22 (.52) | | .23 | 1.43 (.47) | .77 | -1.30 (1.26) | .56 | 1.16 (1.18) | .64 | .38 (.49) | 4.79 (1.15) | 1.91 (.89) | 2.15 (.96) |  |
| Russia | 2.89 (.86) | | .06 (.30) | | .47 | 1.33 (.46) | .85 | -.48 (.95) | .53 | .32 (1.02) | .56 | .58 (.49) | 4.46 (.98) | 2.04 (.89) | 2.18 (.81) |  |
| Rwanda | 3.32 (.69) | | .45 (.66) | | .38 | 1.49 (.45) | .84 | -.62 (1.16) | .49 | -.13 (1.04) | .64 | .35 (.48) | 3.98 (1.34) | 2.71 (.89) | 2.66 (.80) |  |
| Singapore | 3.07 (.81) | | .41 (.70) | | .48 | - | - | -.37 (1.08) | .54 | .38 (1.27) | .55 | .44 (.50) | 4.09 (1.10) | 2.55 (.87) | 2.26 (.89) |  |
| Slovenia | 2.93 (.86) | | .27 (.53) | | .46 | 1.67 (.58) | .84 | -.72 (1.34) | .39 | 1.60 (1.14) | .42 | .51 (.50) | 5.06 (.90) | 1.73 (.72) | 2.16 (.91) |  |

| Variables | | SMIL | | Environmental Engagement | | | | Political Engagement | | | | Openness for change vs. Conservation | | | | Self-transcendence vs. Self-enhancement | | | | Environmental Priority | Importance of Environment | Political Interest | Importance of Politics | |  |
| --- | --- | --- | --- | --- | --- | --- | --- | --- | --- | --- | --- | --- | --- | --- | --- | --- | --- | --- | --- | --- | --- | --- | --- | --- | --- |
| Country/Area | | Mean (SD) | | Mean(SD) | | a | | Mean(SD) | | a | | Mean(SD) | | a | | Mean(SD) | | a | | Mean(SD) | Mean(SD) | Mean(SD) | Mean(SD) | |  |
| South Africa | 3.07 (.88) | | .63 (.83) | | .28 | | 1.56 (.54) | | .81 | | -.05 (.81) | | .77 | | .14 (1.00) | | .74 | | .37 (.48) | | 4.38 (1.18) | 2.44 (1.07) | | 2.45 (1.05) | |
| Zimbabwe | 3.46 (.76) | | .27 (.57) | | .51 | | 1.48 (.52) | | .82 | | -.61 (1.27) | | .34 | | .16 (1.48) | | .43 | | .39 (.49) | | 4.43 (1.28) | 2.38 (1.04) | | 2.32 (1.02) | |
| Spain | 2.76 (.89) | | .16 (.45) | | .42 | | 1.75 (.63) | | .87 | | -.64 (1.27) | | .50 | | - | | .41 | | .38 (.49) | | 4.66 (1.02) | 1.92 (.87) | | 2.03 (.85) | |
| Sweden | 2.95 (.88) | | .57 (.75) | | .36 | | 2.13 (.48) | | .71 | | -.04 (1.44) | | .37 | | 1.47 (1.56) | | .43 | | .66 (.48) | | 4.50 (1.23) | 2.72 (.87) | | 2.66 (.90) | |
| Thailand | 3.17 (.83) | | .38 (.67) | | .46 | | 1.41 (.55) | | .90 | | -.51 (1.02) | | .69 | | .65 (1.13) | | .68 | | .60 (.49) | | 4.40 (1.31) | 3.02 (.86) | | 2.94 (.84) | |
| Trinidad and Tob | 3.69 (.58) | | .42 (.67) | | .43 | | 1.77 (.55) | | .85 | | -1.11 (1.32) | | .30 | | 1.35 (1.39) | | .37 | | - | | 4.84 (1.16) | 2.23 (1.02) | | 2.20 (1.01) | |
| Tunisia | 3.29 (1.02) | | .06 (.29) | | .32 | | 1.31 (.47) | | .84 | | -.83 (1.31) | | .44 | | .02 (1.59) | | .44 | | .36 (.48) | | 4.08 (1.66) | 2.29 (1.11) | | 2.21 (.98) | |
| Turkey | 3.20 (.65) | | .14 (.44) | | .54 | | 1.42 (.54) | | .92 | | -.52 (1.10) | | .47 | | .58 (1.13) | | .46 | | .52 (.50) | | 4.76 (1.02) | 2.47 (.97) | | 2.45 (.87) | |
| Ukraine | 3.05 (.83) | | .10 (.37) | | .47 | | 1.26 (.43) | | .76 | | -1.20 (1.46) | | .50 | | .84 (1.43) | | .55 | | .52 (.50) | | 4.06 (1.27) | 2.07 (.90) | | 2.21 (.88) | |
| Egypt | 3.21 (.92) | | .03 (.20) | | .45 | | 1.08 (.25) | | .71 | | -1.33 (1.35) | | .25 | | .92 (1.13) | | .58 | | .32 (.47) | | 4.63 (1.22) | 2.83 (.96) | | 2.88 (.96) | |
| United States | 3.12 (.81) | | .42 (.75) | | .19 | | 1.96 (.48) | | .72 | | -.78 (1.28) | | .51 | | 1.24 (1.35) | | .55 | | .38 (.49) | | 4.04 (1.28) | 2.57 (.84) | | 2.67 (.95) | |
| Uruguay | 2.96 (1.02) | | .17 (.48) | | .55 | | 1.47 (.54) | | .79 | | -.57 (1.32) | | .44 | | 2.01 (1.48) | | .52 | | .71 (.46) | | 4.61 (1.31) | 2.03 (1.00) | | 1.94 (.99) | |
| Uzbekistan | 3.63 (.62) | | .24 (.58) | | .45 | | - | | - | | -1.33 (1.20) | | .40 | | 1.37 (1.21) | | .42 | | .68 (.47) | | 5.11 (1.04) | 2.38 (1.02) | | 2.34 (.95) | |
| Yemen | 3.17 (.92) | | .08 (.30) | | .54 | | 1.36 (.51) | | .81 | | -1.45 (1.35) | | .30 | | .92 (1.35) | | .50 | | .39 (.49) | | 4.70 (1.17) | 2.31 (1.05) | | 2.32 (.96) | |

Table S2. Correlates of the search for meaning by country and scores of power distance

| Country/Area | SMIL – Pro-environmental engagement | | SMIL – political engagement | | Power distance |
| --- | --- | --- | --- | --- | --- |
|  | *r* | *n* | *r* | *n* |  |
| Algeria | -.03 | 1100 | .16 | 722 | . |
| Argentina | .15 | 948 | .15 | 854 | . |
| Armenia | .07 | 1072 | .04 | 1048 | 49 |
| Australia | .16 | 943 | .12 | 922 | 38 |
| Azerbaijan | -.08 | 1002 | .00 | 1002 | . |
| Belarus | .04 | 1478 |  |  | . |
| Brazil | .04 | 1415 | .06 | 1259 | 69 |
| Chile | .10 | 871 | .05 | 830 | . |
| China | .11 | 1895 | .11 | 1870 | 63 |
| Colombia | .06 | 1466 | .13 | 1435 | 80 |
| Cyprus | .03 | 985 | .12 | 944 | 58 |
| Ecuador | .03 | 1200 | .07 | 1199 | 67 |
| Egypt, Arab Rep. | .02 | 1523 | .04 | 1523 | . |
| Estonia | .06 | 1487 | .05 | 1409 | 78 |
| Georgia | .01 | 1186 | .01 | 1131 | 40 |
| Germany | .13 | 1941 | .11 | 1874 | . |
| Ghana | .01 | 1552 | -.01 | 1552 | . |
| Hong Kong SAR, China | .03 | 960 | .07 | 957 | 35 |
| India | .06 | 4894 | .10 | 4169 | . |
| Iraq | .03 | 1144 | .10 | 1048 | 68 |
| Japan | .11 | 1682 | .12 | 1122 | 77 |
| Jordan | -.06 | 1183 | -.02 | 1176 | . |
| Kazakhstan | .03 | 1500 | .01 | 1500 | 54 |
| Korea, rep. | .10 | 1060 | .16 | 1040 | . |
| Kuwait | .07 | 835 |  |  | . |
| Kyrgyzstan | .06 | 1466 | -.04 | 1464 | 60 |
| Lebanon | -.07 | 1074 | -.02 | 944 | . |
| Libyan Arab Jama | .05 | 1871 | .01 | 1694 | . |
| Malaysia | -.03 | 1299 | -.05 | 1299 | . |
| Mexico | .06 | 1927 | .10 | 1853 | . |
| Morocco | -.07 | 906 | -.05 | 873 | 104 |
| Netherlands | .08 | 1587 | .09 | 1587 | 81 |
| New Zealand | .08 | 668 | .06 | 539 | 70 |
| Nigeria | .01 | 1759 | -.02 | 1759 | 38 |
| Pakistan | .21 | 1174 | .13 | 1143 | 22 |
| Palestine | -.04 | 961 | .04 | 899 | . |
| Peru | .10 | 1151 | .02 | 1020 | 55 |
| Philippines | -.02 | 1192 | -.01 | 1179 | 64 |

Table S2. Correlates of the search for meaning by country (cont’d)

| Country/Area | SMIL – Pro-environmental engagement | | SMIL – political engagement | |  | Power distance |
| --- | --- | --- | --- | --- | --- | --- |
|  | *r* | *n* | *r* | *n* |  |  |
| Poland | *.07* | *917* | *.01* | *842* |  | 68 |
| Qatar | *.07* | *1042* |  |  |  | . |
| Romania | .07 | 1429 | .07 | 1343 |  | 90 |
| Russian Federati | .06 | 2338 | .09 | 2237 |  | 93 |
| Rwanda | .14 | 1527 | .05 | 1527 |  | . |
| Singapore | .10 | 1920 | .11 | 941 |  | 74 |
| Slovenia | .11 | 987 |  |  |  | 71 |
| South Africa | .01 | 3231 | .11 | 2729 |  | . |
| Spain | .06 | 1037 | .15 | 912 |  | . |
| Sweden | .07 | 1116 | .11 | 1056 |  | 57 |
| Taiwan | .13 | 1137 | .12 | 1116 |  | 31 |
| Thailand | .07 | 1097 | .03 | 1094 |  | 64 |
| Trinidad and Tob | .00 | 970 | .03 | 852 |  | 47 |
| Tunisia | .04 | 1086 | .20 | 985 |  | . |
| Turkey | .09 | 1540 | .15 | 1457 |  | 66 |
| Ukraine | .02 | 1500 | .03 | 1500 |  | . |
| United States | .11 | 2112 | .12 | 2088 |  | . |
| Uruguay | .08 | 935 | .22 | 846 |  | 40 |
| Uzbekistan | .06 | 1428 |  |  |  | 61 |
| Yemen | .03 | 920 | .15 | 658 |  | . |
| Zimbabwe | -.02 | 1500 | .04 | 1500 |  | . |

*SMIL = Search for meaning in life*

**Part 2**

1. **Random coefficient models**

Table S3a. Results of random-coefficient models of pro-environmental engagement

| Outcome variable | Pro-environmental Engagement | | | | | |
| --- | --- | --- | --- | --- | --- | --- |
| Individual-Level Moderator | Openness vs. Conservation | | Self-Transcendence vs. Self-Enhancement | | Environmental Priority | Importance of Environment |
| *Level 1 – individual level* |  |  |  |  |  |  |
| Intercept (*β_0_*) | .287 (.023)^***^ | .289 (.023) ^***^ | .285 (.024) ^***^ | .285 (.024) ^***^ | .228 (.023)^***^ | .279 (.023)^***^ |
| **SMIL (***β_1_***)** | **.044 (.006)**^***^ | **.044 (.006)** ^***^ | **.040 (.006)** ^***^ | **.040 (.006)** ^***^ | **.033 (.007)^***^** | **.033 (.006)^***^** |
| Values (*β_2_*) | .039 (.002)^***^ | .039 (.002) ^***^ | .024 (.002) ^***^ | .024 (.002) ^***^ | .094 (.005)^***^ | .050 (.002)^***^ |
| **SMIL x Values (*β_3_*)** | **.010 (.002)**^***^ | **.010 (.002)**^***^ | **-.001 (.002)** | **-.001 (.002)** | **.013 (.006)^*^** | **.002 (.002)** |
| Gender (*β_4_*) | .017 (.004)^***^ | .017 (.004)^***^ | .032 (.005) ^***^ | .032 (.005) ^***^ | .028 (.004)^***^ | .031 (.004)^***^ |
| Age (*β_5_*) | .002 (.000)^***^ | .002 (.000)^***^ | .000 (.000) ^***^ | .000 (.000) ^***^ | .001 (.000)^***^ | .004 (.001)^**^ |
| Education (*β_6_*) | .022 (.001)^***^ | .022 (.001)^***^ | .021 (.001) ^***^ | .021 (.001) ^***^ | .022 (.001)^***^ | .021 (.001)^***^ |
| Income (*β_7_*) | .018 (.001)^***^ | .018 (.001)^***^ | .021 (.001) ^***^ | .021 (.001) ^***^ | .019 (.001)^***^ | .021 (.001)^***^ |
| *Level 2 – societal level* |  |  |  |  |  |  |
| Reliability of values^#^(*γ_01_*) |  | .022 (.021) |  | .-.000 (.023) |  |  |
|  |  |  |  |  |  |  |
| *Residual variance (SE)* |  |  |  |  |  |  |
| Level 1 variance (*r_ij_*) | .367 (.002)^***^ | .367 (.002)^***^ | .373 (.002) ^***^ | .373 (.002) ^***^ | .366 (.002)^***^ | .361(.002)^***^ |
| Level 2 variance of intercept (*u_0j_*) | .031 (.006)^***^ | .030 (.006)^***^ | .030 (.006) ^***^ | .031 (.006) ^***^ | .030 (.006)^***^ | .030 (.006)^***^ |
| **Level 2 variance of slope of search for meaning (*u_1j_*)** | **.002 (.000)**^***^ | **.002 (.000)**^***^ | **.002 (.000)** ^***^ | **.002 (.000)** ^***^ | **.002 (.000)^***^** | **.002 (.000)^***^** |
|  |  |  |  |  |  |  |
| Number of individuals | 77,480 | 77,480 | 74,652 | 74,652 | 74,620 | 80,883 |
| Number of countries | 59 | 59 | 57 | 57 | 58 | 59 |
| Log likelihood | -71249.440 | -71248.931 | -69296.495 | -69296.495 | -68533.445 | -73700.301 |

*Boldface highlights the results of major interest; SMIL = Search for meaning in life; * p < .05; ** p < .01; *** p < .001*

*^#^ To partial out the internal consistency of the scale of openness vs. conservation and that of the scale of self-transcendence vs. self-enhancement, reliabilities of the corresponding scale at the societal level were controlled (see Malka, Soto, Inzlicht, & Lelkes, 2014).*

Table S3b. Results of random-coefficient models of political engagement

| Outcome variable | Political Engagement | | | | | | |  |
| --- | --- | --- | --- | --- | --- | --- | --- | --- |
| Individual-Level Moderator | Openness vs. Conservation | | Self-Transcendence vs. Self-Enhancement | | Political Interest | Importance of Politics | |  |
| *Level 1 – individual level* |  |  |  |  |  |  | |  |
| Intercept (*β_0_*) | 1.475 (.034)^***^ | 1.470 (.034) ^***^ | 1.470 (.035)^***^ | 1.470 (.034)^***^ | 1.480 (.034)^***^ | 1.473 (.034)^***^ | |  |
| **SMIL (***β_1_***)** | **.047 (.006)^***^** | **.047 (.006)^***^** | **.044 (.006)**^***^ | **.044 (.006)**^***^ | **.032 (.005)^***^** | **.039 (.005)^***^** | |  |
| Values (*β_2_*) | .027 (.001)^***^ | .027 (.002)^***^ | .017 (.001)^***^ | .017 (.001)^***^ | .126 (.002)^***^ | .078 (.002)^***^ | |  |
| **SMIL x Values (*β_3_*)** | **.008 (.002)**^***^ | **.008 (.002)**^***^ | **-.001 (.002)** | **-.001 (.002)** | **.0 10 (.002)^***^** | **.013 (.002)^***^** | |  |
| Gender (*β_4_*) | .084 (.004)^***^ | .084 (.004)^***^ | .094 (.004)^***^ | .094 (.004)^***^ | .064 (.004)^***^ | .081 (.004)^***^ | |  |
| Age (*β_5_*) | -.000 (.000)^***^ | -.000 (.000)^***^ | -.001 (.000)^***^ | -.001 (.000)^***^ | -.002 (.000)^***^ | -.001 (.000)^***^ | |  |
| Education (*β_6_*) | .037 (.001)^***^ | .037 (.001)^***^ | .036 (.001)^***^ | .036 (.001)^***^ | .032 (.001)^***^ | .035 (.001)^***^ | |  |
| Income (*β_7_*) | .003 (.001)^**^ | .003 (.001)^**^ | .005 (.001)^***^ | .005 (.001)^***^ | .001 (.001)^***^ | .004 (.001)^***^ | |  |
| *Level 2 – societal level* |  |  |  |  |  | |  | |
| Reliability of values^#^(*γ_01_*) |  | -.042 (.029) |  | -.038 (.031) |  |  | |  |
|  |  |  |  |  |  |  | |  |
| *Residual variance (SE)* |  |  |  |  |  |  | |  |
| Level 1 variance (*r_ij_*) | .226 (.001)^***^ | .226 (.001)^***^ | .224 (.001)^***^ | .224 (.001)^***^ | .214 (.001)^***^ | .214 (.001)^***^ | |  |
| Level 2 variance of intercept (*u_0j_*) | .064 (.012)^***^ | .064 (.012)^***^ | .064 (.013)^***^ | .064 (.012)^***^ | .064 (.012)^***^ | .064 (.012)^***^ | |  |
| **Level 2 variance of slope of search for meaning (*u_1j_*)** | **.001 (.000)**^***^ | **.001 (.000)**^***^ | **.001 (.000)**^***^ | **.001 (.000)**^***^ | **.001 (.000)^***^** | **.001 (.000)**^***^ | |  |
|  |  |  |  |  |  |  | |  |
| Number of individuals | 68,150 | 68,150 | 65,451 | 65,451 | 71,356 | 70,909 | |  |
| Number of countries | 54 | 54 | 52 | 52 | 54 | 54 | |  |
| Log likelihood | -46200.976 | -46199.963 | -44151.525 | -44150.764 | -46498.973 | -47540.206 | |  |

*Boldface highlights the results of major interest; SMIL = Search for meaning in life; * p < .05; ** p < .01; *** p < .001*

*^#^ To partial out the internal consistency of the scale of openness vs. conservation and that of the scale of self-transcendence vs. self-enhancement, reliabilities of the corresponding scale at the societal level were controlled (see Malka, Soto, Inzlicht, & Lelkes, 2014).*

1. **Simple slope effects**

Table S4a. Results of simple slope analysis with pro-environmental engagement as the outcome variable

| Outcome Variable | Pro-environmental Engagement | | | | | |  |
| --- | --- | --- | --- | --- | --- | --- | --- |
| Models with Individual-level value: | Openness vs. Conservation | |  |  | Environmental Priority | |  |
|  | Intercept | Slope of SMIL |  |  | Intercept | Slope of SMIL |  |
| High Power Distance (1 SD above the mean) | .291 (.043)^***^ | .035 (.009)^***^ |  |  | .209 (.042)^***^ | .016 (.010) |  |
| Low Power Distance (1 SD below the mean) | .365 (.039)^***^ | .083 (.008)^***^ |  |  | .293 (.040)^***^ | .075 (.010)^***^ |  |
|  |  |  |  |  |  |  |  |

*SMIL = Search for meaning in life; * p < .05. *** p < .001.*

Table S4b. Results of simple slope analysis with political engagement as the outcome variable

| Outcome Variable | Political Engagement | | | | | |  | | |  |
| --- | --- | --- | --- | --- | --- | --- | --- | --- | --- | --- |
| Models with Individual-level value: | Openness vs. Conservation | |  |  | Political Interest | |  | Importance of Politics | | |
|  | Intercept | Slope of SMIL |  |  | Intercept | Slope of SMIL |  | Intercept | Slope of SMIL | |
| High Power Distance (1 SD above the mean) | 1.351 (.041)^***^ | .038 (.009)^***^ |  |  | 1.357 (.041)^***^ | .023 (.009)^*^ |  | 1.348 (.041)^***^ | .031 (.009)^***^ | |
| Low Power Distance (1 SD below the mean) | 1.746 (.036)^***^ | .066 (.008)^***^ |  |  | 1.750 (.037)^***^ | .049 (.008)^***^ |  | 1.741 (.037)^***^ | .058 (.008)^***^ | |
|  |  |  |  |  |  |  |  |  |  | |

*SMIL = Search for meaning in life; * p < .05. *** p < .001.*

1. **Supplementary analyses**

To exclude the confounding factor of economic development in each society, I conducted a series of supplementary analyses that controlled the GDP per capita of each society, with the information derived from World Bank database (2010). In all the models of pro-environmental engagement, the interaction of the search for meaning and power distance remained significant, suggesting that its effect goes beyond GDP. However, in the models of political engagement, the interaction of the search for meaning and power distance and the interaction of the search for meaning and GDP were both non-significant. These results indicated that the power distance shared a similar effect with GDP in hindering the translation of the search for meaning into political engagement. This is not surprising, considering the large correlation between GDP and power distance (*r* = .64, *p* < .001). This set of supplementary analyses was deemed very stringent, and thus, I mainly relied on the major findings for discussion. Also, for parsimony, it is not advisable to include GDP in the models of political engagement if this does not account for the level 2 variance.

Table S5a. Supplementary analyses results of the models of pro-environmental engagement

| Outcome Variable | Pro-environmental Engagement | | | |
| --- | --- | --- | --- | --- |
| Individual-Level Moderator | Openness vs. Conservation | Self-transcendence vs. Self-enhancement | Environmental Priority | Importance of Environment |
| *Level 1 – Individual level* |  |  |  |  |
| Intercept (*γ_00_*) | .323 (.033)^***^ | .329 (.034)^***^ | .243 (.033)^***^ | .311 (.033)^***^ |
| **SMIL (*γ_10_*)** | **060 (.007)**^***^ | **.059 (.007)**^***^ | **.050 (.008)^***^** | **.046 (.007)**^***^ |
| Values (*β_2_*) | .044 (.003)^***^ | .037 (.002)^***^ | .116 (.006)^***^ | .070 (.002)^***^ |
| **SMIL x Values (*β_3_*)** | **.013 (.003)**^***^ | **-.003 (.003)** | **.016 (.007)^*^** | **.004(.003)** |
| Gender (*β_4_*) | .012 (.006) | .033 (.006)^***^ | .024 (.006)^***^ | .029 (.006)^***^ |
| Age (*β_5_*) | .002 (.000)^***^ | .000 (.000) | .001 (.000)^***^ | .000 (.000)^*^ |
| Education (*β_6_*) | .030 (.002)^***^ | .029 (.002)^***^ | .030 (.002)^***^ | .029 (.001)^***^ |
| Income (*β_7_*) | .013 (.002)^***^ | .016 (.002)^***^ | .015 (.002)^***^ | .017 (.001)^***^ |
| *Level 2 –* *Societal level* |  |  |  |  |
| Power distance (*γ_01_*) | -.035 (.035) | -.038 (.035) | -.036 (.035) | -.035 (.035) |
| GDP (*γ_02_*) | .004 (.043) | -.005 (.044) | .011 (.012) | .006 (.042) |
| *Cross-level interaction* |  |  |  |  |
| **SMIL x Power distance (*γ_11_*)** | **-.026 (.007)**^***^ | **-.027 (.007)**^***^ | **-.033 (.008)^***^** | **-.026 (.007)**^***^ |
| **SMIL x GDP (*γ_12_*)** | **-.004 (.009)** | **-.010 (.009)** | **-.008 (.009)** | **-.005 (.008)** |
|  |  |  |  |  |
| *Residual variance (SE)* |  |  |  |  |
| Level 1 variance (*r_ij_*) | .403 (.003)^***^ | .416 (.003)^***^ | .401 (.002)^***^ | .391 (.003)^***^ |
| Level 2 variance of intercept (*u_0j_*) | .028 (.007)^***^ | .028 (.007)^***^ | .028 (.007)^***^ | .028 (.007)^***^ |
| Level 2 variance of slope of search for meaning (*u_1j_*) | .001 (.000)^*^ | .001 (.000)^*^ | .001 (.000)^**^ | .001 (.000)^*^ |
|  |  |  |  |  |
| Number of individuals | 43,687 | 40,900 | 42,009 | 46,184 |
| Number of countries | 33 | 31 | 32 | 33 |
| Log likelihood | -42243.267 | -40176.461 | -40496.981 | -43921.461 |

*Boldface highlights the results of major interest; SMIL = Search for meaning in life; GDP = Gross domestic product;*

** p < .05; ** p < .01; *** p < .001*

Table S5b. Supplementary analyses results of the models of political engagement

| Outcome Variable | Political Engagement | | | |
| --- | --- | --- | --- | --- |
| Individual-Level Moderator | Openness vs. Conservation | Self-transcendence vs. Self-enhancement | Political Interest | Importance of Politics |
| *Level 1 – Individual level* |  |  |  |  |
| Intercept (*γ_00_*) | 1.53(.026)^***^ | 1.53 (.028)^***^ | 1.530 (.027)^***^ | 1.53 (.027)^***^ |
| **SMIL (*γ_10_*)** | **.051 (.007)** ^***^ | **.050 (.007)**^***^ | **.034 (.007)**^***^ | **.034 (.007)**^***^ |
| Values (*β_2_*) | .038 (.002) ^***^ | .024 (.002)^***^ | .140 (.002)^***^ | .140 (.003)^***^ |
| **SMIL x Values (*β_3_*)** | **.008 (.002)** ^***^ | -.003 (.002) | **.012 (.003)**^***^ | **.017 (.003)**^***^ |
| Gender (*β_4_*) | .069 (.004)^***^ | **.085 (.005)**^***^ | .048 (.005)^***^ | .048 (.005)^***^ |
| Age (*β_5_*) | -.001 (.000) ^***^ | -.001 (.000)^***^ | -.002 (.001)^***^ | -.002 (.000)^***^ |
| Education (*β_6_*) | .047 (.001)^***^ | .046 (.001)^***^ | .039 (.001)^***^ | .040 (.001)^***^ |
| Income (*β_7_*) | .001 (.001) | .003 (.001)^**^ | -.000 (.001) | -.000 (.001) |
| *Level 2 – Societal level* |  |  |  |  |
| Power distance (*γ_01_*) | -.151 (.029)^***^ | -.157 (.029)^***^ | -.150 (.029)^***^ | -.150 (.029)^***^ |
| GDP (***γ_02_***) | .100 (.036)^**^ | .084 (.037)^*^ | .102 (.036)^**^ | .102 (.036)^**^ |
| *Cross-level interaction* |  |  |  |  |
| **SMIL x Power distance (*γ_11_*)** | **-.012 (.007)** | **-.014 (.008)** | **-.011 (.007)** | **-.011 (.007)** |
| **SMIL x GDP (*γ_12_*)** | **.004 (.009)** | **-.002 (.010)** | **.005 (.009)** | **.005 (.009)** |
|  |  |  |  |  |
| *Residual variance (SE)* |  |  |  |  |
| Level 1 variance (*r_ij_*) | .238 (.002)^***^ | .237 (.002)^***^ | .226 (.002)^***^ | .226 (.002)^***^ |
| Level 2 variance of intercept (*u_0j_*) | 018 (.005)^***^ | .018 (.005)^***^ | .018 (.005)^***^ | .018 (.005)^***^ |
| Level 2 variance of slope of search for meaning (*u_1j_*) | .001 (.000)^**^ | .001 (.000)^**^ | .001 (.000)^**^ | .001 (.000)^**^ |
|  |  |  |  |  |
| Number of individuals | 40,134 | 37,462 | 42,723 | 42,190 |
| Number of countries | 32 | 30 | 32 | 32 |
| Log likelihood | -28283.119 | -26286.445 | -28940.273 | -28614.234 |

*Boldface highlights the results of major interest; SMIL = Search for meaning in life; GDP = Gross domestic product;*

** p < .05; ** p < .01; *** p < .001*

**References**

Malka, A., Soto, C. J., Inzlicht, M., & Lelkes, Y. (2014). Do needs for security and certainty predict cultural and economic conservatism? A cross-national analysis. *Journal of Personality and Social Psychology*, *106*(6), 1031-1051.

Snijders T. A. B., & Bosker R. J. (1999). *Multilevel analysis: An introduction to basic and advanced multilevel modeling*. Thousand Oaks, CA: SAGE

World Bank. (2010). GPD per capita (constant 2010 US$): World Bank national accounts data, and OECD National Accounts data files. Retrieved from https://data.worldbank.org/indicator/NY.GDP.PCAP.KD
